# Effects of Dietary Supplementation with a Ferulic Acid-Rich Bioactive Component of Wheat Bran in a Murine Model of Graft-Versus-Host Disease

**DOI:** 10.3390/nu15214582

**Published:** 2023-10-28

**Authors:** Silvia Preciado, Cristina Martínez-Villaluenga, Daniel Rico, Sandra Muntión, María-Carmen García-Macías, Almudena Navarro-Bailón, Ana Belén Martín-Diana, Fermín Sánchez-Guijo

**Affiliations:** 1 Cell Therapy Unit, Hematology Department, University Hospital of Salamanca, Instituto de Investigación Biomédica de Salamanca (IBSAL), University of Salamanca, 37007 Salamanca, Spain; smuntion@usal.es (S.M.); anavarrob@saludcastillayleon.es (A.N.-B.); ferminsg@usal.es (F.S.-G.); 2 RICORS TERAV, ISCIII, 28029 Madrid, Spain; 3 Centro en Red de Medicina Regenerativa y Terapia Celular de Castilla y León, 37007 Salamanca, Spain; 4 Institute of Food Science, Technology and Nutrition (ICTAN-CSIC), José Antonio Novais 10, 28040 Madrid, Spain; c.m.villaluenga@csic.es; 5 Agrarian Technological Institute of Castilla and Leon (ITACyL), Ctra. Burgos Km 119, Finca Zamadueñas, 47071 Valladolid, Spain; ricbarda@itacyl.es (D.R.); mardiaan@itacyl.es (A.B.M.-D.); 6 Department of Medicine and Cancer Research Center, University of Salamanca, 37007 Salamanca, Spain

**Keywords:** wheat bran, ferulic acid, graft-versus-host disease, anti-inflammatory activity, immunomodulation, mouse model

## Abstract

Graft-versus-host disease (GvHD) is a common and severe complication following allogeneic hematopoietic stem cell transplantation (HSCT). Its prevention and treatment is a major challenge. Ferulic acid (FA) has anti-inflammatory and antioxidant properties that could be attractive in this setting. Our aim was to evaluate a bioactive ingredient derived from wheat bran (WB), selected for its high concentration of FA, in a murine model of GvHD. The ingredient was obtained via a bioprocess involving hydrolysis and spray-drying. GvHD was induced via HSCT between MHC-mismatched mouse strains. FA treatment was administered orally. Survival and disease scores (weight loss, hunching, activity, fur texture, and skin integrity, each scored between 0 and 2 depending on disease severity) were recorded daily, histological evaluation was performed at the end of the experiment, and serum inflammatory cytokines were analyzed on days 9 and 28. Treatment with FA did not protect GvHD mice from death, nor did it diminish GvHD scores. However, histological analysis showed that ulcers with large areas of inflammatory cells, vessels, and keratin were less common in skin samples from FA-treated mice. Areas of intense inflammatory response were also seen in fewer small intestine samples from treated mice. In addition, a slight decrease in INF-γ and TNF-α expression was observed in the serum of treated mice on day 28. The results showed some local effect of the ingredient intervention, but that the dose used may not be sufficient to control or reduce the inflammatory response at the systemic level in mice with GvHD. Higher dosages of FA may have an impact when evaluating the immunomodulatory capabilities of the hydrolyzed WB ingredient. Thus, further experiments and the use of technological strategies that enrich the ingredients in soluble ferulic acid to improve its efficacy in this setting are warranted.

## 1. Introduction

Allogeneic hematopoietic stem cell transplantation (allo-HSCT) is a therapeutic procedure that consists of reconstituting hematopoiesis and the immune system via the infusion of hematopoietic stem cells (HSCs) after conditioning treatment with chemo- or radiotherapy. This procedure is often the only potentially curative treatment available for some hematological and non-hematological diseases. Graft-versus-host disease (GvHD) is a prominent and severe complication following allo-HSCT [1]. Immunocompetent donor cells recognize as foreign different epitopes of organs and tissues of the recipient, leading to a massive release of cytokines that trigger inflammatory reactions, primarily affecting the skin, gastrointestinal tract, bone marrow, and liver [2,3]. The first-line treatment of GvHD involves the use of steroids and prophylactic immunosuppressive therapy. However, there is no standard second-line treatment when the disease persists. In light of this, numerous immunosuppressive strategies have been employed, including extracorporeal photopheresis, ruxolitinib, calcineurin inhibitors, mycophenolate mofetil, and rapamycin, among others [4]. Mesenchymal stem cells (MSCs) have also been used to treat GvHD, and some MSC-based advanced therapy medical products have been approved in some countries and are commercially available [5,6].

In addition to the effects induced by GvHD, the gastrointestinal tract can also be affected by the toxicity of the conditioning regimen, leading also to diarrhea, mucositis, or digestive infections that often lead to malnutrition and weight loss. Since a variety of bioactive dietary components have been shown to influence the immune system, the development of nutraceuticals enriched in immunomodulatory and antioxidant compounds could be a potential adjuvant to standard therapy in order to prevent or treat intestinal GvHD and associated tissue damage.

Nutraceuticals have received considerable attention due to their potential benefits in improving health status. Cereal brans contain polyphenols that may exert antioxidant and immunomodulatory effects [7]. Polyphenols occur in different forms in cereal brans, including aglycones (free ferulic acids), esters, glycosides, and/or bound polymerized complexes. In particular, ferulic acid (FA), the main phenolic compound in wheat bran (WB) [8,9], is mostly bound to cell wall polysaccharides and proteins, resulting in limited bioavailability. To improve the bioaccessibility, bioavailability, and ultimate bioactivity of this compound, our group has developed new technological strategies involving enzymatic hydrolysis combined with thermomechanical treatments. These approaches are designed to increase the ratio of free to bound FA content in WB, improving also their antioxidant and anti-inflammatory properties in vitro [10,11].

FA could be an interesting compound in efforts to reverse the toxicity of GvHD via several biochemical mechanisms due to its anti-inflammatory properties. One of them is its antioxidant capacity, reducing oxidative stress, free radicals, and reactive oxygen species (ROS). Ferulic acid also inhibits the production of pro-inflammatory cytokines such as IL-1β, IL-6 and tumor necrosis factor-alpha (TNF-α). Additionally, it may also regulate the activity of immune cells, such as macrophages and neutrophils, involved in the inflammatory response. Finally, it has been shown that FA may exert an angiogenic effect through HIF-1α [12,13,14,15,16,17,18]. In fact, its therapeutic capacity has already been explored in Alzheimer’s disease, arthritis or diabetes [19,20,21,22]. In addition, FA has demonstrated radioprotective properties, although its mechanism of action is not well understood, and it plays an important role as a detoxifying agent in the liver [23]. Both free and polymerized forms of FA can be absorbed in the stomach of rodents, while only free forms can be absorbed in the small intestine. The absorption of FA is known to increase with oral administration [24].

With all this background, this study aims to address the hypothesis that the intake of dietary supplements enriched in free FA may potentially reduce chronic inflammation as well as the degree of oxidative stress associated with GvHD, thus contributing to post-transplant immunotolerance. If this hypothesis is corroborated, we could consider these compounds as potential dietary adjuvants to prophylactic immunosuppressor therapy or to standard therapy for GvHD. Therefore, in the current study, our aim is to evaluate the effect of a bioactive ingredient derived from hydrolyzed WB, selected for its elevated concentration of free FA, as treatment in a well-established murine model of GvHD [25,26].

The originality of this work lies in the fact that it is the first study of the biological and clinical effects of an ingredient rich in FA on a severe murine model of GvHD.

## 2. Materials and Methods

### 2.1. Ingredient Preparation

The ingredient tested was obtained using a bioprocess from WB following the method previously described by Martín-Diana and Tomé-Sánchez [8,10]. Briefly, 100 g of WB was hydrated in deionized water (1:20, *w:v*) and autoclaved at 115 °C, 1.2 × 10^5^ Pa for 15 min (Ilpra Plus 100; Ilpra Systems, Barcelona, Spain). The treated sample was hydrolyzed using Ultraflo XL enzyme at 1% (enzyme:wheat bran ratio, *w:w*) and incubated at 47 °C, 20 h, in a controlled water bath using a continuous magnetic stirrer at 1000 rpm [9]. Enzyme reaction was heat-inactivated. WB supernatant fraction was collected and spray-dried using an MMbasic rotary system (GEA Mobile MinorTM, Düsseldorf, Germany) at 6 × 10^2^ Pa with a flow rate of 0.78 L/h and an air inlet/outlet temperature of 130/85 °C. The powered ingredient was stored at 4–8 °C until further use.

### 2.2. Generation of a GvHD Murine Model

Eight- to ten-week-old C57BL/6 (H-2Kb) and BALB/c (H-2Kd) mice were obtained from Charles River Laboratories (Barcelona, Spain) and maintained at the Animal Care Facility of the University of Salamanca for two weeks before the start of the experiments. All procedures followed the Spanish and European Union guidelines (RD 1201/05 and 86/609/CEE) and were approved by the Bioethics Committee of the University of Salamanca (reg. 0000593). Animals were housed under specific pathogen-free conditions with ad libitum access to food and water. All experiments were performed under the same controlled and standardized conditions of room temperature, airflow, and humidity.

The murine GvHD model was established as previously reported, albeit with minor modifications [25]. C57BL/6 mice were used as donors and BALB/c (H2d) female mice functioned as recipients. Prior to transplantation, recipient mice were irradiated with a lethal dose of 800 cGy total body irradiation (TBI) and divided in two fractions using a cesium source (Gammacell-200, Nordion International). We intravenously injected 10 × 10^6^ of allogeneic donor bone marrow cells with or without 4 × 10^6^ splenocytes as a source of T cells through the tail vein. Four groups were established: 1—mice receiving only TBI (TBI mice); 2—mice with TBI and receiving only allogeneic bone marrow cells (BM mice); 3—mice with TBI and GvHD (receiving allogeneic bone marrow and splenocytes) (GvHD mice); and 4—mice with TBI, GvHD and FA treatment (GvHD + ferulic acid mice). FA treatment was administered daily via oral gavage in a volume of 200 µL containing 100 mg of the spray-dried hydrolyzed WB ingredient. The daily dose of FA was 10.85 mg per kg mouse body weight.

A total of 36 mice were used in this work: 2 TBI mice, 2 BM mice, 16 GvHD mice, and 16 GvHD + ferulic acid mice.

### 2.3. GvHD Scoring

Disease severity was assessed as described by Cooke et al. [27]. Five clinical parameters were scored at a rate of three days per week: weight loss, posture (hunching), activity, fur texture, and skin integrity. Depending on the severity of the disease symptoms, each parameter was scored between 0 (no disease symptoms) and 2 (indicating the highest severity). A clinical index was calculated by adding the individual scores of the five criteria. Mice survival was also recorded daily. This allowed us to assess whether or not the treatment was effective.

### 2.4. Histopathological Analysis of GvHD

Histological evaluation of each experimental group was performed on day 28 after transplantation. Mice were sacrificed and GvHD target organs were isolated: skin, small intestine, liver, and bone marrow. Tissue samples were then fixed in 4% paraformaldehyde at room temperature for approximately 48 h, embedded in paraffin, sectioned at 3 µm thickness, and stained with hematoxylin-eosin (H&E) for evaluation according to standard protocols. Slides were examined by an experienced pathologist under a light microscope in a blinded fashion.

### 2.5. Cytokines Profiles Analysis

On days 9 and 28 after transplantation, serum soluble inflammatory cytokines were analyzed using cytometric bead array (CBA). For this purpose, peripheral blood was obtained from mice by puncture in the mandibular vein (3–4 drops) and kept for 30 min to allow coagulation. After centrifugation, the supernatant (serum) was collected and frozen at −80 °C until further analysis. The “BD Cytometric Bead Array (CBA) Mouse Th1/Th2/Th17 Cytokine Kit” (Beckton Dickinson, San Diego, CA, USA) was used to simultaneously evaluate some soluble murine cytokines interleukin (IL)-6, IL-17A, IL-10, IL-2, IL-4, interferon (INF)-γ and tumor necrosis factor (TNF)-α, following the manufacturer’s instructions. Samples were acquired in a FACS Canto flow cytometer (BD Biosciences, Franklin Lakes, NJ, USA) and analyzed using the kit’s own software.

### 2.6. Statistical Analysis

Statistical analysis was performed with GraphPad Prism 7.0 software (GraphPad Software, Inc., San Diego, CA, USA). Data are presented as mean ± standard error of the mean (SEM) or median with interquartile range. The Mann–Whitney test was used for statistical comparison, and differences were considered statistically significant when *p*-values were <0.05. Survival was analyzed using Kaplan–Meier survival curves and the log-rank (Mantel–Cox) test.

## 3. Results

### 3.1. Survival and Clinical GvHD Score

**Figure 1:**
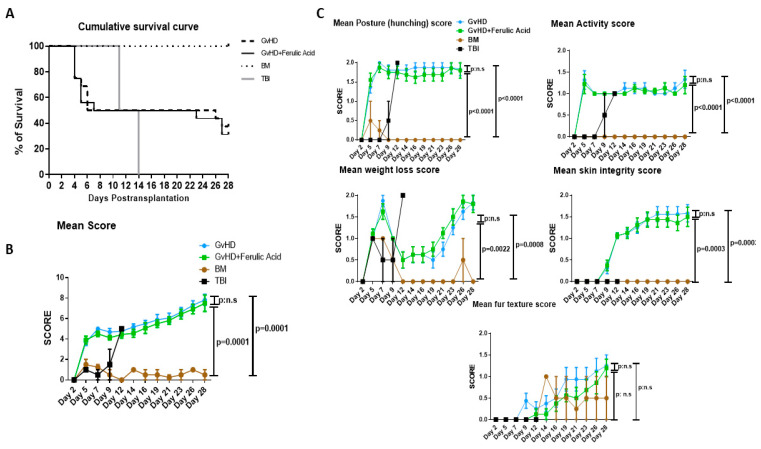
Clinical GvHD. (**A**) Survival after transplantation. Overall survival of different experimental groups: total body irradiation (TBI; *n* = 2), BM group (*n* = 2), GvHD group (*n* = 16), and GvHD + ferulic acid (*n* = 16) were represented in a Kaplan-Meier curve and analyzed using the Mantel–Cox log-rank test. Data are represented as percentage of survival (**B**) GvHD clinical score. Animals were regularly scored for five clinical parameters (posture, activity, weight loss, skin integrity, and fur texture) on a scale from 0 to 2. Clinical GvHD score was generated by summation of these five parameters. Data are represented as mean ± SEM. (**C**) Each of the five clinical parameters (posture, activity, weight loss, skin integrity, and fur texture) scored separately on a scale from 0 to 2. Data are represented as mean ± SEM. BM: bone marrow; GvHD: graft-versus-host disease; TBI: total body irradiation; SEM: standard error of the mean.

The long-term survival and GvHD score of recipient mice were determined after daily FA treatment. The control group, BM mice receiving only donor BM cells without any additional intervention, survived for 28 days, recovering their hematopoietic system after transplantation. The group received only total body irradiation died between days 10–14. The survival of the GvHD group, which received BM and spleen cells, was 37% on day 28 after transplantation. Treatment with FA did not protect GvHD mice from death, with survival in this group even lower at 31%. Differences between groups were not significant when assessed via long-rank analysis of survival. Maximum mortality was observed around days 4–6 after transplantation and then after day 22 in both GvHD and GvHD + FA groups (Figure 1A).

Mice were evaluated and scored three days per week for body weight loss, posture (hunching), activity, fur texture, and skin integrity. Individual scores were summed up to produce the clinical score (Figure 1B). As expected, no signs of GvHD were observed in the BM group. Their score remained below 2 throughout the experiment, but a slight weight loss in the first week was detected due to the irradiation conditioning. TBI mice receiving only irradiation suffered from hunched posture, severe weight loss, and decreased activity from day 7 of the experiment and had to be sacrificed between days 10 and 14. Co-transplantation of splenocytes into GvHD mice induced GvHD symptoms with an increase in all individual scores. Specifically, mice displayed low activity, a hunched posture, loss of fur, and damaged skin (Figure 1C). Hunching, decrease in activity, and weight loss became importantly altered by days 5–7, while skin integrity was altered later around days 12–14 after transplantation. FA treatment did not improve GvHD symptoms, nor did it lead to lower GvHD scores (Figure 1B).

### 3.2. Histological Evaluation of Murine GvHD

**Figure 2:**
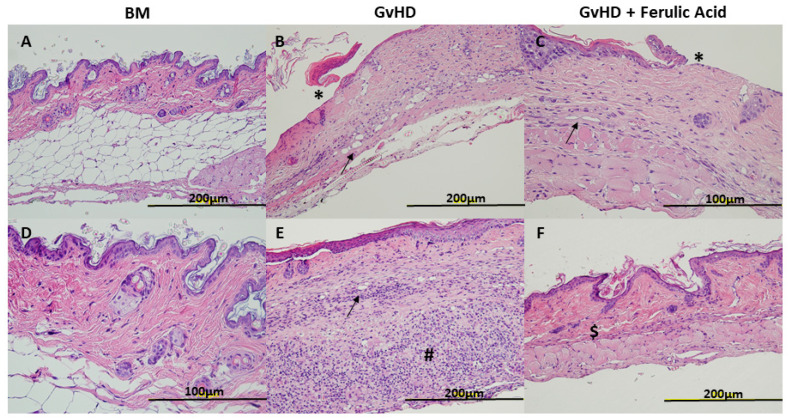
Morphology of skin tissue sections. Representative images of skin tissue in BM mice, GvHD mice, and GvHD + ferulic acid mice stained with H&E 28 days after transplantation at 20× magnification (**A**,**B**,**E**,**F**), and 40× magnification (**C**,**D**). BM: bone marrow; GvHD: graft-versus-host disease. Arrows indicate vessel increase, * indicates ulcers with associated queratin, # represents important areas of inflammation, and $ represents fibrosis.

On day 28 after transplantation, hematoxylin-eosin (H&E) staining was performed on tissues from each experimental group, and slides were examined by an experienced pathologist (Figure 2). We isolated one sample of each tissue per mouse, so each mouse was assessed using its representative sample from each tissue. Regarding skin histology, GvHD mice showed a thinned epidermis in more than half of the cases, sometimes with ulcers and wounds, where ulcers are defined as any discontinuity or open sore in the epidermis (40% of the samples). As compared to control mice, a thinner dermis and an absent hypodermis without hair follicles were observed in most GvHD samples. In some cases, an increase in inflammatory cells, vessels, and keratin was also observed, usually in areas where the epidermis was disrupted, in wounds (Figure 2B,E). In the case of mice treated with hydrolyzed WB rich in free FA, the changes observed were similar. More than 50% of the samples showed a thinned epidermis. However, ulcers with increased inflammatory cells, vessels, and keratin were present in fewer samples (20%), and a generally thinner dermis and an absent hypodermis were observed (Figure 2C,F).

**Figure 3:**
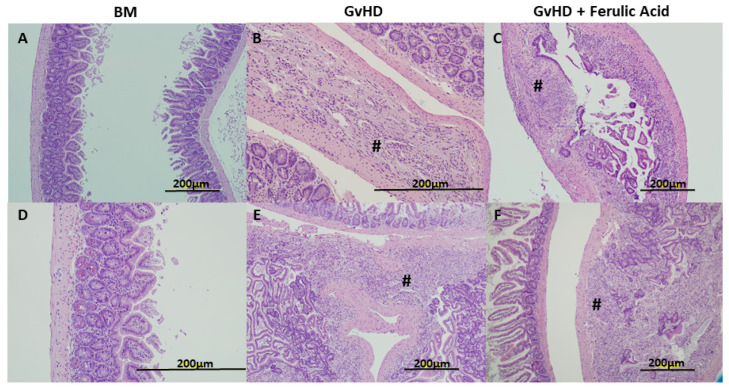
Morphology of small bowel tissue sections. Representative images of small bowel tissue in BM mice, GvHD mice, GvHD + FA mice stained with H&E 28 days after transplantation at 10× magnification (**A**,**C**,**E**,**F**) and 20× magnification (**B**,**D**). BM: bone marrow; GvHD: graft-versus-host disease. **#** points out significant inflammatory reaction sites with crypt loss.

The histology of small intestinal tissue samples showed a significant alteration in the GvHD mice, with small areas of inflammation along the gastrointestinal tract with polymorphonucleated cells in all samples. However, significant inflammatory reactions with cellular infiltration in the tissue, and increases in neutrophils, lymphocytes, macrophages and other inflammatory cells leading to a loss of submucosa and mucosa were also observed in 60% of the samples analyzed (Figure 3B,E). In treated mice, similar small areas of inflammation were found in some samples, and important inflammation reactions were observed in only 40% of samples (Figure 3C,F).

**Figure 4:**
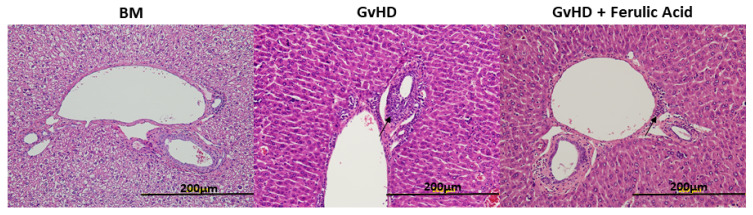
Morphology of liver tissue sections. Representative images of liver tissue in BM mice, GvHD mice, and GvHD + ferulic acid mice stained with H&E 28 days after transplantation at 20× magnification (**A**–**C**). BM: bone marrow; GvHD: graft-versus-host disease. Arrows indicate inflammatory reaction sites.

In liver tissue samples, the alterations were not so evident; we found some thickened canaliculi with more connective tissue, small inflammatory reactions, and more fibroblasts. These small changes were observed in both treated and untreated mice (Figure 4).

**Figure 5:**
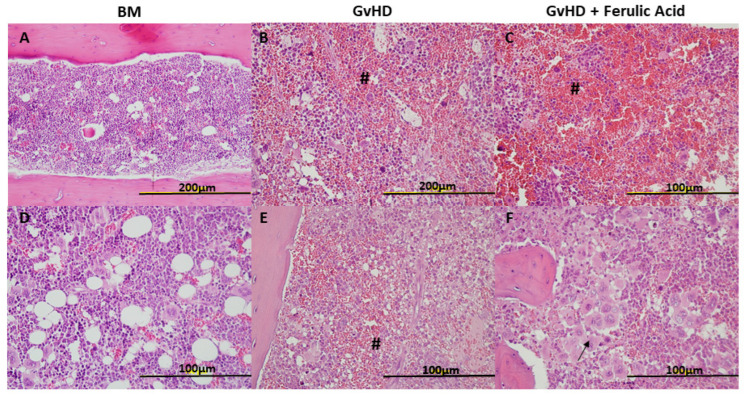
Morphology of bone marrow tissue sections. Representative images of bone marrow tissue in BM mice, GvHD mice and GvHD + ferulic acid mice stained with H&E 28 days after transplantation. (**A**), 20× magnification. (**B**–**F**), 40× magnification. BM: bone marrow; GvHD: graft-versus-host disease. Arrows point out megakaryocytes, # represents hemorrhagic areas.

Finally, an increase in the number of erythrocytes was observed in the BM, generally in all GvHD group samples, treated and untreated, with some hemorrhagic areas in many samples. In non-hemorrhagic areas, the BM cellularity was normal, except for one sample of treated mice, where the percentage of megakaryocytes was quite high (Figure 5).

### 3.3. Evaluation of Murine Serum Cytokine Profile

**Figure 6:**
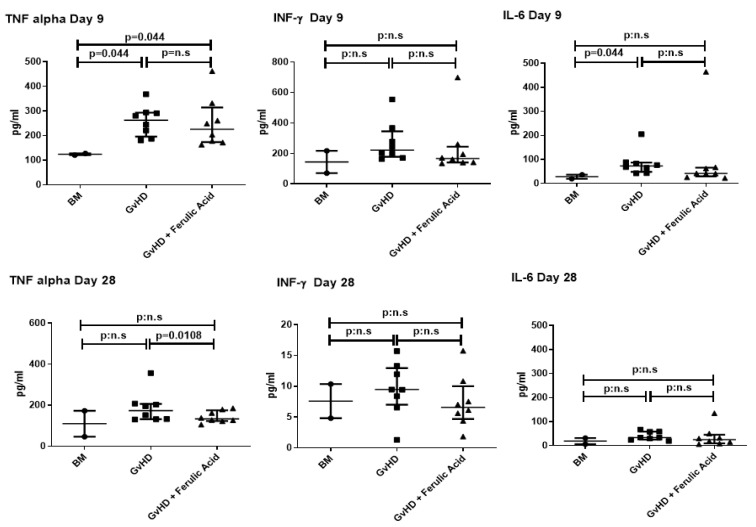
Analysis of cytokines in serum from bone marrow (BM)-transplanted mice, mice with GvHD, and mice with GvHD treated with ferulic acid. Serum levels of TNF-α, INF- γ, and IL-6 were analyzed on days 9 and 28 after BMT. The data represent medians with interquartile ranges. BM: bone marrow; GvHD: graft-versus-host disease.

Serum-soluble inflammatory cytokines were analyzed on days 9 and 28 after transplantation. TNF-α and IL-6 were significantly overexpressed in mice with GvHD than in controls at day 9. After FA treatment, TNF-α expression was significantly reduced (Figure 6).

**Figure 7:**
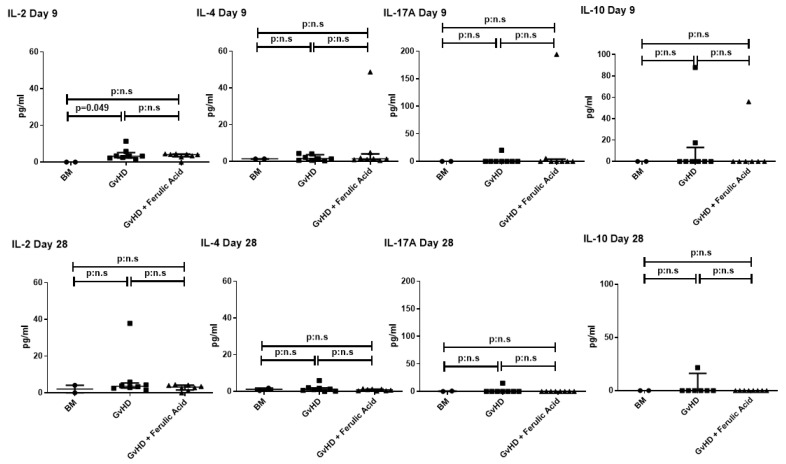
Analysis of cytokines in serum from bone marrow (BM)-transplanted mice, mice with GvHD, and mice with GvHD treated with ferulic acid. Serum levels of IL-2, IL-4, IL-17A, and IL-10 were analyzed on days 10 and 28 after BMT. Data are represented as median with interquartile ranges. BM: bone marrow; GvHD: graft-versus-host disease.

IL-2 and IL-4 had very low expression in all experimental groups and showed no differences between treated and untreated mice, although IL-2 was overexpressed in GvHD compared to control mice at day 9. Finally, IL-17A and IL-10 were not expressed in most of the samples, and there were no significant differences among groups (Figure 7).

## 4. Discussion

The current manuscript is the first study investigating the biological and clinical effects of an ingredient rich in FA on a severe murine model of GvHD. Several studies have previously assessed the immunomodulatory effects of various natural components derived from food or plants in this setting. Certain phenolic compounds—such as polyphenolic extract from olive oil or resveratrol, another natural polyphenolic compound—can reduce T-cell activation and proliferative capacity in vitro [28]. In addition, they are capable of reducing the release of pro-inflammatory cytokines. Notably, resveratrol administration can reduce the severity of collagen-induced arthritis in mice. Furthermore, in GvHD mice, an increase in survival rates and a decrease in GvHD scores are observed when mice are treated with a diet supplemented with phenolic extracts [28,29]. Curcumin and epigallocatechin-3-gallate (EGCG), a polyphenol found in green tea, exhibited antitumor properties by inhibiting the PI3K/AKT pathway [30,31]. EGCG also attenuates GvHD in mice by reducing the proliferative capacity of donor T cells and blocking specific cell surface molecules [32,33].

In this regard, our previous study demonstrated the potential immunomodulatory and antioxidant properties of hydrolyzed WB, an excellent source of free FA capable of modulating the immune system. Hydrolyzed WB was produced through the optimization of novel strategies to yield the highest concentration of soluble phenolic compounds, thereby maximizing the antioxidant and anti-inflammatory potential of the WB. Our previous studies have shown a reduction in TNF-α production in LPS-induced murine macrophages in the presence of this WB ingredient and an increased trend in most of the antioxidant parameters [8,9,10,11]. Other studies have also demonstrated, both in vitro and in vivo, the anti-inflammatory effects of FA. It has been reported that administration of FA modulates dendritic cells and ameliorates inflammation in murine models of asthma. In rats with collagen-induced arthritis, the administration of FA has been shown to reduce serum levels of IL-1β and TNF- α as well as apoptosis and the expression of pro-inflammatory cytokines in diabetic rats [12,13,20,21,34]. Moreover, it has been demonstrated how FA can restore intestinal dysbiosis and barrier dysfunction, mitigating tissue damage in LPS-challenged chickens [35]. In this sense, FA can prevent the distortion of occluding and E-cadherin proteins after heat stress-induced epithelial barrier dysfunction [36]. Taken together, these investigations support the notion that FA may have robust anti-inflammatory and therapeutic properties in GvHD mice.

However, in contrast to other studies in which GvHD mice were treated with anti-inflammatory compounds, we observed no discernible differences in either survival or GvHD scores between mice treated daily with 10.85 mg/kg of FA or untreated mice [29,32,37]. It is true that some compounds, such as EGCG, show disparities because the increase in survival is minimal and variable depending on the dose. Discrepancies become apparent when the dose reaches at least 25 mg/kg [32,33,38]. In most inflammatory murine models, doses between 10 mg/kg and 100 mg/kg have been used as once-daily injections [33]. In fact, the dose of pure FA that leads to improvements in the gut health of LPS-challenged chickens is 100 mg/kg [35].

In the context of histological analysis, when we assessed small intestinal tissues, it became apparent that significant inflammatory sites accompanied by mucosal and submucosal loss appeared in 60% of untreated mice, whereas they were present in only 40% of treated mice. To evaluate the presence and extent of inflammatory sites in histologic samples, we assessed cellular infiltration, that is to say the increase in or presence of immune cells, such as neutrophils, lymphocytes, and macrophages, in the tissue. Considering small inflammatory sites those with high number of these immune cells and significant inflammatory sites those in which, in addition, the tissue architecture was completely lost due to the amount of inflammatory cells (absence of cripts). In line with these data, some groups described a decrease in tissue damage after treatment with immunomodulatory compounds in GvHD mice as a reduction in the number of apoptotic bodies per crypt [29] or in the number of crypt abscesses and degree of crypt loss in the colon [33].

Similarly, in skin samples, GvHD mice treated with the hydrolyzed WB had fewer wound areas, fewer areas of increased vascularity, and reduced inflammatory features. Comparable results were observed in skin following EGCG treatment [33]. In contrast, treatment with polyphenolic extracts did not result in improvements in skin histological analysis [29].

Consistent with the effects published in mice treated with olive oil polyphenolic extract or EGCG, we observed a slight reduction in INF-γ and a significant reduction in TNF-α levels in mice treated with hydrolyzed WB. This reduction is important because these cytokines represent two of the three primary mediators in the GvHD cytokine storm, and substantial release of pro-inflammatory cytokines is directly correlated with tissue damage [39]. However, we did not observe differences in the expression of IL-2 and IL-17, as they are barely expressed, always below 10 pg/mL, even in the untreated GvHD mice. Conversely, in studies with polyphenolic extracts or EGCG, these cytokines were expressed in the GvHD mice and are reduced only in treated mice [29,32].

The results showed some local effect of the hydrolyzed WB intervention but that the dose of treatment used may not be sufficient to produce a systemic effect in this severe acute GvHD murine model. Therefore, additional investigations involving higher dosages of FA will be essential in order to comprehensively evaluate the immunomodulatory capabilities of the hydrolyzed WB ingredient. To this end, it is necessary to continue working on technological strategies to enrich the ingredient in soluble ferulic acid to improve its bioefficacy in this setting.

## 5. Conclusions

Dietary supplementation with our bioactive ingredient, derived from wheat bran and selected for its high concentration of ferulic acid, showed some local effects, such as reductions in damage and inflammation in small intestine and skin histological samples and slight decreases in INF-γ and TNF-α expression. However, the dose used may not be sufficient to control or reduce the inflammatory response at the systemic level in mice with GvHD.

## Data Availability

The raw data supporting the conclusion of this article will be made available by the authors, without undue reservation.
